# Covid-19 no Brasil: Aprendendo a Andar no Escuro sem Deixar Nada para Trás

**DOI:** 10.36660/abc.20200445

**Published:** 2020-06-29

**Authors:** Juliano Lara Fernandes

**Affiliations:** 1 Radiologia Clínica de Campinas CampinasSP Brasil Radiologia Clínica de Campinas, Campinas, SP - Brasil; 2 Instituto de Ensino e Pesquisa Jose Michel Kalaf CampinasSP Brasil Instituto de Ensino e Pesquisa Jose Michel Kalaf,Campinas, SP – Brasil

**Keywords:** Coronavirus, COVID-19, Pandemia, Doença Catastrófica/mortalidade, Hospitalização/economia, Equidade no Acesso aos Serviços de Saúde, Testes Diagnósticos/métodos, Equipamento de Proteção Individual, Máscaras, Ventiladores Mecânicos

*“Se Você Tem Altos e Baixos, Celebre, pois Significa que está Vivo!”*

A pandemia pelo SARS-CoV-2 teve seu primeiro caso no Brasil em 04/02/2020 no estado de São Paulo. Como um verdadeiro meteoro, praticamente paralisou o planeta sem que tivéssemos uma medida terapêutica mais efetiva para combater o vírus além de práticas utilizadas globalmente apenas décadas ou até centenas de anos atrás, com nível de evidência moderna muito pouco robusta.^1^ O Brasil teve a vantagem de antevisão da pandemia por estar algumas semanas atrás dos seus pares asiáticos e europeus, podendo identificar erros e acertos desses países na sua preparação para enfrentar o problema.

Assim, descobrimos meio por acaso que temos o terceiro maior número de leitos de UTI do mundo, perdendo apenas para EUA e Alemanha,^2^ mas também nos deparamos com enorme heterogeneidade entres estados, limitação e burocracia para aquisição de equipamentos de proteção individual e kits de testagem. Isso fez com que algumas opções de abordagem da pandemia adotadas em outros países não pudessem ser reproduzidas aqui, seja pela impossibilidade de um *lockdown* num país continental, seja pela agilidade e custos necessários para se fazer a identificação dos casos transmissores. Além disso, a limitação do número de testes a serem realizados nos deixou sem um guia preciso sobre o momento em que a pandemia se encontrava, dificultando muito o planejamento para alocação eficiente dos escassos recursos de forma correta e no tempo certo.

A solução para isso foi adotar estratégias próprias que permitissem caminharmos no escuro, mas tendo ao menos uma ideia mais próxima sobre em que altura do caminho estávamos. Tal informação é essencial para decisões importantes que impactam não só a parte econômica do país, mas também toda a cadeia de saúde da população, que se viu privada de acessos ambulatoriais e tratamentos eletivos por conta da pandemia. Os modelos matemáticos epidemiológicos teóricos se mostraram pouco capazes de predizer nossos números reais, seja por superestimar a letalidade da doença que parece se comportar mais próximo de 0.2-0.5%,^3^ seja por usar dados de pandemias passadas com outras dinâmicas de transmissão. Interessantemente, os modelos que mais se prestaram a modelar em que altura da pandemia estamos vieram de estratégias de ajuste de curvas ou modelos bayseanos utilizando dados já existentes em outros países ou com base nos dados preliminares que já tínhamos em nossas curvas.^4 , 5^ Esses modelos apareceram de forma relativamente espontânea e fora de grupos de pesquisa tradicionais, mas se revelaram mais assertivos em determinar diversos momentos da pandemia.^6^

De forma mais simplificada, mas também utilizando dados de como a pandemia se comportou previamente em outros países, buscamos analisar os gráficos de casos novos por dia de 30 países com os maiores números de casos de Covid-19, de acordo com os seguintes critérios: países que já haviam atingido um pico máximo e apresentavam ao menos 5 dias de queda ou estabilização dos casos novos diários.^7^ A China foi excluída pois concentrou num dia posterior milhares de casos anteriores; Brasil foi excluído por ser objeto da aplicação do resultado. Foram incluídos 5 países que não tiveram isolamento mandatório. Dos 30 países, 18 já foram considerados como tendo concluído o ciclo completo da pandemia, com número de casos novos diários <70% do pico de casos novos por dia. Utilizando a data do 1º, 100º e 200º caso ou 10º^o^ óbito, foram determinados os tempos entre essas datas e o pico de casos novos/dia da doença. A partir desses resultados, observou-se que 95% dos países estudados apresentavam seus picos 55±8 dias do 1º caso, 31±5 dias do 100º caso, 27±5 dias do 200º caso e 19±4 dias do 10º óbito. Com esse dado, seria possível se estabelecer qual seriam os picos de casos novos, óbitos e uso do sistema hospitalar em diversos estados e cidades do país, mesmo sem se ter a certeza do número exato de casos pela subnotificação, com base no comportamento da pandemia em países com os mais distintos sistemas de saúde e medidas de mitigação do vírus. As datas de pico de óbitos foram estabelecidas a partir de 14 dias do pico de casos e do uso do sistema hospitalar a partir de 26 dias do pico de casos, tendo em vista os tempos de incubação, manifestação de sintomas, internação e eventual piora clínica da doença.^8^ Com essas premissas, estimamos os diversos picos em cada um dos estados brasileiros com os maiores números de casos, mostrados na Tabela 1 (limitado apenas à previsão a partir do 100º caso para exemplificar o modelo). O que não se consegue prever com nenhum desses modelos é a parte descendente da curva, mais rápida em alguns locais e muito mais lenta — exibindo um platô — em outros, demandando leitos hospitalares por tempo mais prolongado, um possível efeito de virulência distinta do vírus após inúmeras mutações.^9^

**Tabela 1:** – Previsão de picos estaduais de casos novos/dia, óbitos e uso hospitalar baseado em modelagem de 30 países (mostrado apenas com estimativa a partir do 100º caso). Dados estimados para efeito de pesquisa, pendentes de modificação e verificação

Estado	1º Caso	100º Caso	200º Caso	10º Óbito	Pico 100º caso	IC95% inf.	IC95% sup	Pico Óbitos (100)	Pico Hospitalar (100)
SP (Reg Metrop)	04/fev	02/mar	06/mar	06/mar	02/abr	28/mar	06/abr	16/abr	28/abr
CE	14/fev	07/mar	10/mar	15/mar	07/abr	02/abr	11/abr	21/abr	03/mai
GO	02/mar	08/mar	11/mar	26/mar	08/abr	03/abr	12/abr	22/abr	04/mai
SC	28/fev	14/mar	17/mar	04/abr	14/abr	09/abr	18/abr	28/abr	10/mai
RJ	27/fev	15/mar	18/mar	18/mar	15/abr	10/abr	19/abr	29/abr	11/mai
DF	26/fev	15/mar	18/mar	04/abr	15/abr	10/abr	19/abr	29/abr	11/mai
BA	26/fev	16/mar	19/mar		16/abr	11/abr	20/abr	30/abr	12/mai
RN	08/mar	18/mar	21/mar	07/abr	18/abr	13/abr	22/abr	02/mai	14/mai
RS	09/mar	21/mar	25/mar	08/abr	21/abr	16/abr	25/abr	05/mai	17/mai
MG	17/mar	23/mar	27/mar	02/abr	23/abr	18/abr	27/abr	07/mai	19/mai
MT	19/mar	24/mar	27/mar	26/abr	24/abr	19/abr	28/abr	08/mai	20/mai
PR	12/mar	26/mar	01/abr	06/abr	26/abr	21/abr	30/abr	10/mai	22/mai
AM	18/mar	28/mar	01/abr		28/abr	23/abr	02/mai	12/mai	24/mai
PE	12/mar	02/abr	05/abr	01/abr	03/mai	28/abr	07/mai	17/mai	29/mai
MA	20/mar	05/abr	07/abr	06/abr	06/mai	01/mai	10/mai	20/mai	01/jun
PA	18/mar	06/abr	10/abr	11/abr	07/mai	02/mai	11/mai	21/mai	02/jun
PB	19/mar	11/abr	17/abr	09/abr	12/mai	07/mai	16/mai	26/mai	07/jun
AL	10/mar	17/abr	21/abr	18/abr	18/mai	13/mai	22/mai	01/jun	13/jun
PI	19/mar	17/abr	21/abr	18/abr	18/mai	13/mai	22/mai	01/jun	13/jun

*IC: intervalo de confiança*

Mas se estamos acostumados a sempre nos guiar por números mais precisos na cardiologia, como saber se as estimativas estão corretas e checar se não desviamos dos números reais? Para isso, buscamos nas fontes oficiais dados que nos permitem inferir e conferir esses cálculos. Infelizmente, ao menos até esta data da pandemia, criou-se uma confusão tremenda na forma de divulgação de dados que fez com que a interpretação da fase da pandemia no país fosse muito prejudicada. Devido aos atrasos na verificação das infecções pelo SARS-CoV-2, muitos casos foram notificados com dias e até semanas de atraso, fazendo com que os órgãos oficiais soltassem números de registros confirmados como números de ocorrências reais no dia, confundindo os boletins de imprensa e causando muitas vezes alarmes desnecessários, sobretudo quando números acumulados de finais de semana e feriados eram anunciados nas terças-feiras na compilação atrasada.^10^ Para tentar então entender os números, é necessário tentar checar fontes diversas com números ajustados e, sobretudo, buscar na informação dos óbitos uma estatística um pouco mais realista do que ocorre no país, dado que essa métrica é muito mais robusta do ponto de vista de notificação apesar de refletir o que se passou 14 dias atrás. Neste sentido, destaco aqui a importante contribuição dos dados registrados no Portal da Transparência, organizado pela Associação Nacional de Registradores de Pessoas Naturais, que permite um acompanhamento mais acurado do número de óbitos por Covid-19 ou suspeitos na data real da ocorrência e não do registro.^11^ Junto com esse dado, as informações de monitorização das internações por Síndromes Respiratórias Agudas Graves através do sistema InfoGripe também auxiliaram a contínua monitorização das tendências e confirmação ou não das previsões realizadas.^12^

Apesar de todas essas ferramentas à disposição, as medidas implantadas de isolamento social foram tomadas de forma bastante controversa, muitas vezes não se entendendo a fase do ciclo da pandemia em que estávamos, com adoção tardia e às vezes seguindo um curso adotado sem apresentar dados sólidos que justificassem as medidas tomadas. Dado a grande diferença entre estrutura de recursos encontrada no país e fases da pandemia em cada estado, certamente os graus de isolamento deveriam ser bastante distintos visto que cada medida individualmente ou em conjunto tem reflexos diferentes na redução da transmissão viral. Neste sentido, devemos também lembrar do princípio de Pareto, onde 20% do que fazemos atinge 80% do resultado: a aplicação correta de um distanciamento social bem feito, com redução de 25% do original, permite a manutenção da resposta eficaz de transmissão uma vez reduzido inicialmente o R0.^13^ Logo, medidas relativamente simples de ensino da população de lavagem de mãos, distanciamento, máscaras, etc., sendo bem aplicadas, podem muitas vezes ser superior a tentativas de medidas mais drásticas mas executadas de forma desorganizada e sem o entendimento da população onde ela é feita sem preparo adequado.

A decisão do que fazer e em que momento da pandemia atuar é crucial para que não transformemos o objetivo de salvar vidas numa frase apenas apelativa com resultados consolidados que resultam em mais mortes que vidas salvas. Os efeitos de segunda e terceira ordem que ocorrem em qualquer terapia muitas vezes podem ser mais nocivos que o próprio tratamento, especialmente quando este é executado sem o devido planejamento. Muito comum em situações onde uma centralização tenta simplificar processos extremamente complexos e que envolvem cadeias múltiplas (como temos num exemplo famoso da fabricação de um simples lápis),^14^ o efeito final pode ser exatamente o oposto do que perseguimos. E aqui temos várias situações onde um prolongamento desnecessário de medidas de confinamento podem levar a maior número de mortes que pela própria doença. No momento em que este artigo é escrito, o número de óbitos no país pela Covid-19 é de cerca de 10.000 pacientes e temos quase 45 dias de medidas de isolamento. Devido à uma redução prevista de 30% de angioplastias primárias, aumento de tempos de transferência e reinternações por síndromes coronárias agudas não atendidas,^15 - 17^ estima-se um excesso de mortes cardiovasculares de mais de 3.000 mortes neste período. Perdas de consultas ambulatoriais de diversas especialidades elevam o risco de óbitos em até 1.5x, somando a este excesso outras 9.000 mortes desnecessárias.^18^ O aumento de 1% do desemprego ou queda do Produto Interno Bruto por si só estão associados a aumento de óbitos de até 1.63x na população economicamente ativa, adicionando a essa soma mais 3.500 óbitos em excesso.^19^ Cálculos de mortalidade por falha de terapias para neoplasias e perdas diagnósticas no Brasil ainda não existem mas, nos EUA e Inglaterra, as mortes em excesso foram calculadas em 34.000 e 6.000, respectivamente.^20^ Todas essas mortes estão associadas a diversas causas, mas sobretudo pela falta de acesso em tempo aos sistemas de saúde sobrecarregados e focados apenas numa causa de mortalidade. Esses serão os óbitos invisíveis da pandemia e do isolamento por não terem sido previstos como efeitos secundários às medidas unifocais.

O que virá nas próximas semanas e meses está além da visão aqui proposta e vai depender em muito de como vamos decidir sair desta crise sanitária, mais cedo ou de forma mais tardia. Os gastos já altos com as doenças crônicas provavelmente vão aumentar muito nos próximos meses,^21^ sobrecarregando um governo já com excesso de dívida. O desemprego e queda de renda levarão muitos brasileiros a migrarem para um Sistema Único de Saúde já inchado e com a demanda reprimida nestes meses. Ao mesmo tempo, outras soluções que aumentem a eficiência do sistema vão ser aprimoradas como já foram neste curto espaço de tempo, como receitas digitais e telemedicina.^22^ Esse aumento de produtividade médica pode aliviar em parte esses aumentos de demanda e custos, fazendo com que nosso sistema de saúde consiga demonstrar a resiliência e efetividade que fez com que, apesar de todas as dificuldades, tenhamos uma mortalidade por milhão até 10x menor que outros países europeus na mesma fase da doença. O que sabemos é que estaremos prontos para novos desafios, pois foram sempre os otimistas que nos surpreenderam ao mostrar como a inventividade humana é capaz de se sobrepor a obstáculos.
