# Supplementary material for: Covid-19 no Brasil: Aprendendo a Andar no Escuro sem Deixar Nada para Trás
Source: Arq Bras Cardiol. 2020 Jun 29;114(6):988–91. [Article in Portuguese] doi: 10.36660/abc.20200445 (PMC8416130; doi:10.36660/abc.20200445)
Supplement: Translation

# Translation: Covid-19 in Brazil: Learning How to Walk in the Dark Without Leaving Anything Behind

**Authors:** Juliano Lara Fernandes

**Affiliations:** 1 Brazil Radiologia Clínica de Campinas, Campinas, SP - Brazil; 2 Brazil Instituto de Ensino e Pesquisa Jose Michel Kalaf, Campinas, SP – Brazil

**Keywords:** Coronavirus, COVID-19, Pandemics, Catastrófica Illness/mortality, Hospitalization/economics, Equity in Access to Health Services, Diagnostic Tests/methds, Personal Protective Equipment, Masks, Ventilators Mechanical

*“If you have ups and downs, celebrate it because it means you’re alive!”*

The SARS-CoV-2 pandemic had its first case in Brazil on February 4, 2020 in the state of São Paulo. Like a real meteor, it practically paralyzed the planet without a more effective therapeutic approach to fight the virus, in addition to practices adopted worldwide only decades or even hundreds of years ago, with a very low level of robust modern evidence.^1^ Brazil had the advantage of previewing the pandemic as it was a few weeks behind its Asian and European peers, so it was able to identify the hits and failures of these countries in their preparation to face the problem.

We have then found out by chance that we have the world’s third biggest number of ICU beds, second only to the USA and Germany,^2^ but we are also faced with an enormous heterogeneity between states, limitations and bureaucracy for the purchase of personal protective equipment and testing kits. This has prevented some pandemic approaches taken by other countries from being adopted here, either due to the impossibility of a lockdown in a continental country, or due to the speed and costs to identify the cases of transmission. Besides, limitations on the number of tests to be done has left us without a precise guide as to the spread of the pandemic, making it difficult to plan the timely, orderly and efficient allocation of scarce resources.

The solution to that was to adopt our own strategies, which would allow us to walk in the dark while at least having a better idea of how far along the path we were. This information is essential for important decisions impacting not only the economic scenario of Brazil, but also the entire healthcare chain of a population that has been deprived of access to outpatient services and elective treatments due to the pandemic. Theoretical epidemiological mathematical models proved to be little capable of predicting our real figures, either because they overestimate the lethality of the disease that seems to stand closer to 0.2–0.5%,^3^ or because they are based on data from past pandemics with other transmission dynamics. Interestingly, the models that were most suitable for modeling at what point in the pandemic we are resulted from curve adjustment strategies or Baysean models using data from other countries or based on the preliminary data that we already had on our curves.^4 , 5^ These models appeared relatively spontaneously and outside traditional research groups, but proved to be more assertive in determining different moments of the pandemic.^6^

In a simpler way, but also using data on how the pandemic has previously progressed in other countries, we analyzed the charts of new cases per day from 30 countries with the highest number of covid-19 cases according to the following criteria: countries had reached a peak and presented at least 5 days of decline or stabilization of new daily cases.^7^ China was excluded as it concentrated thousands of previous cases on a later day; Brazil was excluded because it was subject to the application of the result. Five countries that did not have mandatory isolation were included. Of the 30 countries, 18 were considered to have completed the full pandemic cycle, with a number of new daily cases <70% of the peak of new cases per day. Using the date of the 1^st^, 100^th^and 200^th^case or 10^th^death, the times between these dates and the peak of new cases/day were determined. From these results, it was found that 95% of the countries studied had their peaks 55±8 days from the 1^st^case, 31±5 days from the 100^th^case, 27±5 days from the 200^th^case and 19±4 days from the 10^th^death. With this data, it would have been possible to establish the peaks of new cases, deaths and use of the hospital system in different states and cities in Brazil, even without being sure of the exact number of cases due to underreporting, based on the behavior of the pandemic in countries with different healthcare systems and mitigation measures. Death peak dates were established after 14 days of peak of cases and use of the hospital system after 26 days of peak of cases, considering incubation times, manifestation of symptoms, hospitalization and potential clinical worsening.^8^ With these criteria, we estimated the different peaks in each of the Brazilian states with the highest number of cases, as shown in Table 1 (limited only to the forecast from the 100^th^case to exemplify the model). What cannot be predicted with any of these models is the descending portion of the curve, faster in some places and much slower, showing a plateau in others, requiring hospital beds for longer times, a potential virulence effect that is different from the virus after many mutations.^9^

**Table 1:** – Prediction of state peaks of new cases/day, deaths and hospital use based on modeling from 30 countries (shown only with an estimate from the 100th case). Estimated data for research purposes, pending modification and verification

State	1^st^Case	100^th^Case	200^th^Case	10^th^Death	Peak 100^th^Case	Lower 95%CI	Upper 95%CI	Death Peaks (100)	Hospital Peak (100)
SP (Metrop. Area)	Feb 4^th^	Mar 2^nd^	Mar 6^th^	Mar 6^th^	Apr 2^nd^	Mar 28^th^	Apr 6^th^	Apr 16^th^	Apr 28^th^
CE	Feb 14^th^	Mar 7^th^	Mar 10^th^	Mar 15^th^	Apr 7^th^	Apr 2^nd^	Apr 11^th^	Apr 21^st^	May 3^rd^
GO	Mar 2^nd^	Mar 8^th^	Mar 11^th^	Mar 26^th^	Apr 8^th^	Apr 3^rd^	Apr 12^th^	Apr 22^nd^	May 4^th^
SC	Feb 28^th^	Mar 14^th^	Mar 17^th^	Apr 4^th^	Apr 14^th^	Apr 9^th^	Apr 18^th^	Apr 28^th^	May 10^th^
RJ	Feb 27^th^	Mar 15^th^	Mar 18^th^	Mar 18^th^	Apr 15^th^	Apr 10^th^	Apr 19^th^	Apr 29^th^	May 11^th^
DF	Feb 26^th^	Mar 15^th^	Mar 18^th^	Apr 4^th^	Apr 15^th^	Apr 10^th^	Apr 19^th^	Apr 29^th^	May 11^th^
BA	Feb 26^th^	Mar 16^th^	Mar 19^th^		Apr 16^th^	Apr 11^th^	Apr 20^th^	Apr 30^th^	May 12^th^
RN	Mar 8^th^	Mar 18^th^	Mar 21^st^	Apr 7^th^	Apr 18^th^	Apr 13^th^	Apr 22^nd^	May 2^nd^	May 14^th^
RS	Mar 9^th^	Mar 21^st^	Mar 25^th^	Apr 8^th^	Apr 21^st^	Apr 16^th^	Apr 25^th^	May 5^th^	May 17^th^
MG	Mar 17^th^	Mar 23^rd^	Mar 27^th^	Apr 2^nd^	Apr 23^rd^	Apr 18^th^	Apr 27^th^	May 7^th^	May 19^th^
MT	Mar 19^th^	Mar 24^th^	Mar 27^th^	Apr 26^th^	Apr 24^th^	Apr 19^th^	Apr 28^th^	May 8^th^	May 20^th^
PR	Mar 12^th^	Mar 26^th^	Apr 1^st^	Apr 6^th^	Apr 26^th^	Apr 21^st^	Apr 30^th^	May 10^th^	May 22^nd^
AM	Mar 18^th^	Mar 28^th^	Apr 1^st^		Apr 28^th^	Apr 23^rd^	May 2^nd^	May 12^th^	May 24^th^
PE	Mar 12^th^	Apr 2^nd^	Apr 5^th^	Apr 1^st^	May 3^rd^	Apr 28^th^	May 7^th^	May 17^th^	May 29^th^
MA	Mar 20^th^	Apr 5^th^	Apr 7^th^	Apr 6^th^	May 6^th^	May 1^st^	May 10^th^	May 20^th^	Jun 1^st^
PA	Mar 18^th^	Apr 6^th^	Apr 10^th^	Apr 11^th^	May 7^th^	May 2^nd^	May 11^th^	May 21^st^	Jun 2^nd^
PB	Mar 19^th^	Apr 11^th^	Apr 17^th^	Apr 9^th^	May 12^th^	May 7^th^	May 16^th^	May 26^th^	Jun 7^th^
AL	Mar 10^th^	Apr 17^th^	Apr 21^st^	Apr 18^th^	May 18^th^	May 13^th^	May 22^nd^	Jun 1^st^	Jun 13^th^
PI	Mar 19^th^	Apr 17^th^	Apr 21^st^	Apr 18^th^	May 18^th^	May 13^th^	May 22^nd^	Jun 1^st^	Jun 13^th^

*CI: confidence interval.*

But if we are used to being guided by more precise figures in cardiology, how do we know if the estimates are correct and if we have been drifting away from the actual figures? We have then looked for official sources that could allow us to infer and check these calculations. Unfortunately, at least until this date of the pandemic, there has been tremendous confusion regarding data reporting, which has caused the interpretation of the pandemic phase in Brazil to be greatly impaired. Due to delayed determination of SARS-CoV-2 infections, many cases have been reported days and even weeks late, causing the official authorities to report numbers of confirmed cases as numbers of actual daily cases, confusing press bulletins and often causing unnecessary fuss, especially when accumulated numbers of weekends and holidays were late announced on Tuesdays.^10^ In order to try to understand the numbers, it is necessary to check different sources with adjusted numbers and, above all, to look for more realistic statistics on the information on deaths occurring in the country, as this metric is much more robust from the point of view of reporting despite reflecting what happened 14 days prior. In this regard, it is worth mentioning the important contribution of the data reported on the Transparency Portal organized by the National Association of Registrars of Vital Statistics, which allows a more accurate monitoring of the number of deaths from Covid-19 or suspected deaths on the actual date of the occurrence, rather than on the reporting date.^11^ Along with this data, information on monitoring hospitalizations for Serious Acute Respiratory Syndromes through the InfoGripe system also helped to continuously monitor trends and confirm or not the forecasts made.^12^

Despite all these tools at hand, the social isolation measures were taken in a very controversial way, often not considering the stage of the pandemic cycle we were at, with a late adoption, and sometimes following a course of action without presenting consistent data that would justify the measures taken. Given the great difference between the structure of resources found in the country and the phases of the pandemic in each state, the degrees of isolation should certainly be quite different since each measure individually or together has different effects on reducing viral transmission. In this sense, we must also remember the Pareto principle, where 20% of what we do reaches 80% of the result: the correct application of well-done social distancing, with 25% reduction from the original distancing, allows an effective transmission response to be maintained once R0 is initially reduced.^13^ Therefore, relatively simple measures of advising the population to wash their hands, keeping away from others, wearing masks, etc., as long as they are well applied, can often be better than attempts at taking drastic yet disorderly actions not well understood by the population concerned and without proper preparation.

The decision of what to do and at what point in the pandemic is crucial so that we do not turn the goal of saving lives into a merely appealing pitch with consolidated results that result in more deaths than lives saved. The second and third order effects that occur in any therapy can often be more harmful than the treatment itself, especially when it is carried out without proper planning. Very common in situations where centralization tries to simplify extremely complex processes that involve multiple chains (as we have in a famous example of pencil making),^14^ the final effect can be exactly the opposite of what we are pursuing. And here we have several situations where an unnecessary extension of confinement measures can lead to a greater number of deaths than from the disease itself. As of writing this, the number of deaths from Covid-19 in Brazil is about 10,000 patients and we have almost 45 days of isolation. Due to an expected 30% reduction in primary angioplasty surgeries, increased transfer times and rehospitalizations due to untreated acute coronary syndromes,^15^ an excess of cardiovascular deaths of over 3,000 is estimated in this period. Missing outpatient visits of various specialties increases the risk of death by up to 1.5 times, adding to this excess another 9,000 unnecessary deaths.^18^ The 1% increase in unemployment or the drop in Gross Domestic Product alone is associated with an increase in deaths of up to 1.63 times in the economically active population, adding another 3,500 excess deaths to this amount.^19^ Mortality calculations due to failure of therapies for neoplasms and misdiagnoses in Brazil do not yet exist, but in the USA and England, excess deaths were estimated at 34,000 and 6,000 respectively.^20^ All of these deaths are associated with several causes, mainly due to the lack of timely access to overburdened healthcare systems solely focused on one cause of mortality. These will be the invisible deaths of the pandemic and isolation because they were not predicted as the side effects to single-point measures.

What will come in the next weeks and months is beyond the views expressed here and will depend, to a great extent, on how we will choose to defuse this health crisis, sooner or later. Expensive spending on chronic diseases is likely to increase dramatically in the coming months,^21^ overburdening a government already in debt. Unemployment and declining income will lead many Brazilians to migrate to a Public Healthcare System already burdened from the demand repressed over these months. At the same time, other solutions to increase the efficiency of the systems will be improved as they have been in this short period of time, such as digital prescriptions and telemedicine.^22^ This increase in medical productivity can partly alleviate these increases in demand and costs, making our healthcare system to demonstrate the resilience and effectiveness that has made us to witness a mortality per million up to 10 times less than other European countries at the same stage of the disease, despite all our difficulties. What we do know is that we will be ready for new challenges, as optimists have always surprised us as to how human inventiveness is capable of overcoming obstacles.
